# An Unusual Pure Periorbital Electrical Injury: A Case Report

**DOI:** 10.29252/wjps.10.2.107

**Published:** 2021-05

**Authors:** Gholamreza Motazedian, Ali Khojasteh

**Affiliations:** 1 Department of Plastic and Reconstructive Surgery, Shiraz University of Medical Sciences, Shiraz, Iran

**Keywords:** Periorbital electrical injury, Water dispenser

## Abstract

Pure periorbital electrical injuries are uncommonly reported and may cause both immediate and delayed complications. These injuries are rare and pose a difficult challenge for both ophthalmologist and plastic surgeon. Here we report an unusual case of pure periorbital electrical injury in a 12-yr old boy while drinking water from water dispenser.

## INTRODUCTION

In patients with severe burn usually the face will be affected and facial burn lesions will lead to psychological and physical morbidity. Ocular and periorbital injuries are reported in 20% facial thermal burns^1^. Future prognosis of periorbital burn injury depends on duration of exposure, mechanism of injury, tissue damage, quality of treatment and risk of infection^2^.

## CASE REPORT

**Fig. 1:**
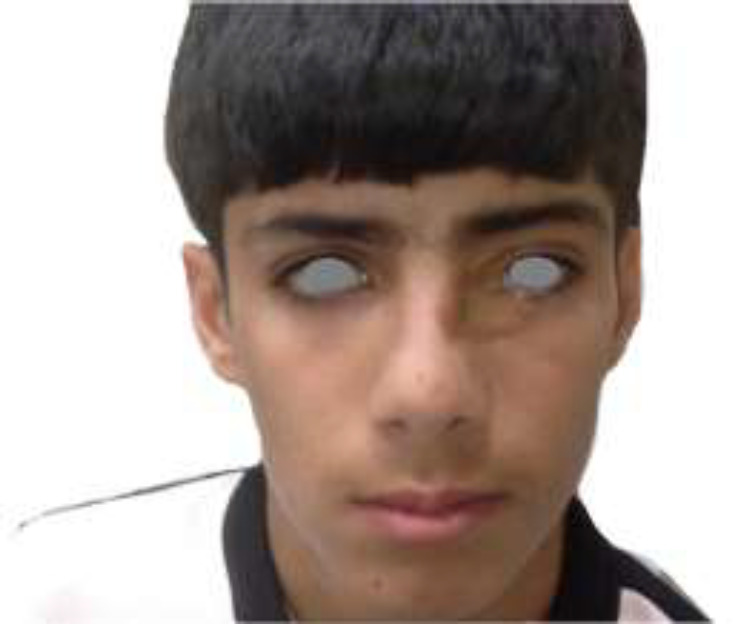
A 12-year old boy with pure periorbital electrical injury at medial side of left periorbital region and left medial canthus while drinking water from water dispenser

**Fig. 2:**
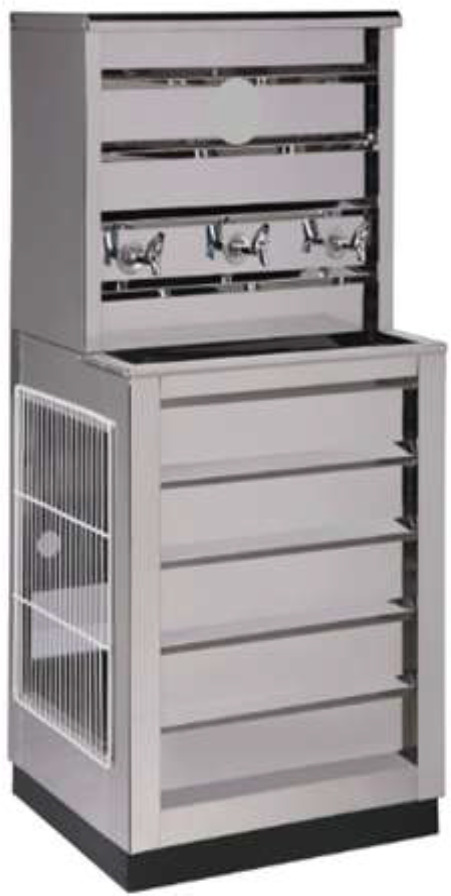
water dispenser

A 12-year old boy referred to our clinic in Shiraz University of Medical Sciences, Shiraz, Iran with deep electrical burn at medial side of left periorbital region and left medial canthus. He was accidentally electrocuted while drinking water directly from water dispenser in the school yard (Figure 1). On examination he had involvement of upper and lower medial left eyelids and left medial canthus. There was no sign and symptom of globe injury. First under general anesthesia debridement of the wound was done and then full thickness skin graft harvested from left posterior auricular area and used for coverage of defects. The reported case had a rapid and complete healing thanks to the timely and appropriate treatment (Figure 2).

**ETHICAL APPROVAL**

Informed consent was obtained from this patient. All procedures performed in this patient were in accordance with the ethical standards of the institutional and/or national research committee and with the 1964 Helsinki declaration and its later amendments or comparable ethical standards.

## DISCUSSION

Electricity is a serious environmental and health hazard especially in developing countries^3^. Patients with extensive burn require fluid resuscitation upon admission and in the emergency phase patients with facial burn should be fully examined to rule out periorbital injuries^4^. Patients with severe facial burn often have large total body surface areas burned and therefore require a prolonged intensive care unit course with periods of medical instability that may prevent timely return to the operating room. In addition, these patients may require prolonged periods of sedation that may inhibit their ability to protect their own corneas.

Management of burned eyelids can provide challenges in both acute and reconstructive burn periods. Suboptimal eyelid burn management can result in potentially devastating complications including severe eyelid contracture, ectropion, corneal ulceration, and even vision loss^5^^,^^6^. Current eyelid management protocols include aggressive lubrication in the days following injury and timely excision and skin grafting with thick split-thickness or full-thickness skin grafts^7^. In addition, aggressive treatment of ectropion with repeated grafting is also part of modern eyelid burn protocols^8^.

The reported case had a rapid and complete healing thanks to the timely and appropriate treatment.

## CONCLUSION

Pure electrical periorbital injury is an uncommon event. It is important to note that by taking simple measures electrical eye injuries are preventable. Therefore holding a good public awareness program not only can save eyes but also lives.
